# Structure of AMH bound to AMHR2 provides insight into a unique signaling pair in the TGF-β family

**DOI:** 10.1073/pnas.2104809118

**Published:** 2021-06-21

**Authors:** Kaitlin N. Hart, William A. Stocker, Nicholas G. Nagykery, Kelly L. Walton, Craig A. Harrison, Patricia K. Donahoe, David Pépin, Thomas B. Thompson

**Affiliations:** ^a^Department of Pharmacology and Systems Physiology, University of Cincinnati, Cincinnati, OH 45267;; ^b^Department of Molecular Genetics, Biochemistry, and Microbiology, University of Cincinnati, Cincinnati, OH 45267;; ^c^Department of Physiology, Monash University, Clayton, VIC 3800, Australia;; ^d^Department of Chemistry and Biotechnology, Swinburne University of Technology, Hawthorn, VIC 3122, Australia;; ^e^Pediatric Surgical Research Laboratories, Massachusetts General Hospital, Boston, MA 02114;; ^f^Department of Surgery, Harvard Medical School, Boston, MA 02115

**Keywords:** anti-Müllerian hormone, Müllerian inhibiting substance, anti-Müllerian hormone receptor type II, structure, transforming growth factor beta

## Abstract

Anti-Müllerian hormone (AMH), or Müllerian-inhibiting substance, is a protein hormone that promotes Müllerian duct regression during male fetal sexual differentiation and regulation of folliculogenesis in women. AMH is a member of the transforming growth factor beta (TGF-β) family, which has evolved to signal through its own dedicated type II receptor, AMH receptor type II (AMHR2). Structures of other TGF-β family members have revealed how ligands infer specificity for their cognate receptors; however, it is unknown how AMH binds AMHR2 at the molecular level. Therefore, in this study, we solved the X-ray crystal structure of AMH bound to the extracellular domain of AMHR2 to a resolution of 2.6Å. The structure reveals that while AMH binds AMHR2 in a similar location to Activin and BMP ligand binding to their type II receptors, differences in both AMH and AMHR2 account for a highly specific interaction. Furthermore, using an AMH responsive cell-based luciferase assay, we show that a conformation in finger 1 of AMHR2 and a salt bridge formed by K534 on AMH and D81/E84 of AMHR2 are key to the AMH/AMHR2 interaction. Overall, our study highlights how AMH engages AMHR2 using a modified paradigm of receptor binding facilitated by modifications to the three-finger toxin fold of AMHR2. Furthermore, understanding these elements contributing to the specificity of binding will help in the design of agonists or antagonists or the selection of antibody therapies.

## Significance

Anti-Müllerian hormone (AMH) plays a crucial role in male sex differentiation and female reproductive development. As such, AMH is widely used as a biomarker for measuring a woman’s fertility, estimating onset of menopause, and has been implicated in reproductive syndromes such as polycystic ovarian syndrome and premature ovarian failure. Despite its biological relevance, how AMH functions on the molecular level is not well understood. In this study, we show that AMH engages its receptor, AMHR2, using an extensive interface distinct from other type II receptors. Furthermore, we identify several regions in both AMH and AMHR2 that are responsible for specificity and required for AMH signaling.

Anti-Müllerian hormone (AMH) or Müllerian-inhibiting substance (MIS) was first identified in the late 1940s to play a critical role in male sex differentiation as the factor responsible for regression, the Müllerian duct (1). During fetal development, the presence of AMH causes regression of the Müllerian ducts, the anlagen of the female reproductive tract, while male sex steroids enhance differentiation of the Wolffian duct into the male reproductive tract. Mutations in AMH or its receptor AMH receptor type II (AMHR2) are linked to the rare genetic syndrome termed persistent Müllerian duct syndrome (PMDS), in which the Müllerian duct only partially regresses (2, 3). As a result, males are born with normal male reproductive organs but additionally develop a uterus and Fallopian tubes from the remaining portions of the Müllerian duct. More recently, it has been shown that AMH plays a fundamental role in the regulation and progression of follicles within the female ovary (4). Specifically, overexpression of AMH in the ovary can suppress early folliculogenesis (5). As such, AMH is being considered as a possible contraceptive and as a biotherapeutic to halt folliculogenesis during chemotherapy, in order to preserve follicles for subsequent cycles (4–6).

AMH is a member of the transforming growth factor beta (TGF-β) family, which consists of over 30 unique, secreted ligand dimers, divided into three classes based on sequence identity and receptor utilization: TGF-βs, bone morphogenetic proteins (BMPs), and Activins (7). To signal, ligands within this family bind the small extracellular domains (ECDs) of two type I and two type II serine/threonine kinase receptors. Upon formation of the heterotetrameric complex, the constitutively active type II receptor will phosphorylate the type I receptor, which in turn phosphorylates intracellular Smad proteins. Phosphorylated Smad proteins then accumulate in the nucleus to regulate gene transcription (8). Interestingly, despite the large number of ligands in the family, there are only seven type I receptors (Alks1 to Alks7) and five type II receptors (Tβ-R2, BMPR2, ActRIIA, ActRIIB, and AMHR2). This receptor bottleneck means that certain ligands will signal using common receptors or combinations of receptors. Generally, members of the TGF-β class utilize Tβ-R2, whereas Activin and BMP ligands utilize BMPR2, ActRIIA, and ActRIIB. One distinction occurs for the affinities of the ligands with their various receptors. While TGF-β and Activin ligands bind to their type II with high affinity (*K*_d_ low nanomolar) and the type I receptors with low affinity (*K*_d_ > 1 μM), BMP ligands typically bind to the type I receptor with higher affinity than the type II receptor (9). For the TGF-β class, type I receptor affinity is enhanced via a type II–type I interaction that is ligand dependent (10). Remarkably, AMH has evolved within this family to be the only member with its own dedicated type II receptor, AMHR2. Affinity measurements indicate that AMH is similar to the Activin and TGF-β class, in which the type II receptor, AMHR2, is the high-affinity receptor (11).

All TGF-β family ligands are dimeric and have a structurally conserved growth factor–like fold that adopts a butterfly shape, from a side view, or a propeller-like shape, from a top view, with a distal convex surface and proximal concave surface formed at the dimer interface. Over the years, crystallographic studies have illuminated how the ECDs of the TGF-β type I and type II receptors bind their cognate ligands (7, 9). All ligands generally bind the type I receptor at the convex dimer interface, while more striking differences occur upon type II receptor binding. For TGF-βs, type II receptor binding occurs at the extreme end or fingertips on each ligand, whereas the type II receptors for BMP and Activin ligands are centrally shifted, binding to the convex surface or knuckle region (7). Despite their differences in ligand binding, these structures revealed that the ECDs of the type I and type II receptors are structurally conserved and consist of a single ECD, with five conserved anti-parallel beta sheets, that adopts a canonical three-finger toxin fold (7). Structures are now available for four of the five type II receptors (ActRIIA, ActRIIB, BMPR2, and Tβ-R2), leaving AMHR2 as the only type II receptor without a solved structure (10, 12–18). While these studies helped define the molecular contacts in both the ligand and receptor across the TGF-β family that are important for receptor specificity, the molecular basis for how AMHR2 selectively binds to AMH has not been resolved.

Previous modeling and mutagenesis studies have suggested that AMH binds the type II receptors, similar to BMP and Activin ligands, on the knuckle region (19, 20). However, these studies are limited in that they do not provide a basis for how AMH selectively binds and signals through AMHR2. To help resolve this question, we solved the crystal structure of the mature C terminus of AMH in complex with the ECD of AMHR2. The structure provides a foundation for understanding AMH/AMHR2 specificity and reveals important elements in both the receptor and ligand that form unique interactions not present within the other type II receptors or TGF-β ligands.

## Results

### Crystal Structure of AMH Bound to AMHR2 ECD.

**Fig. 1.**
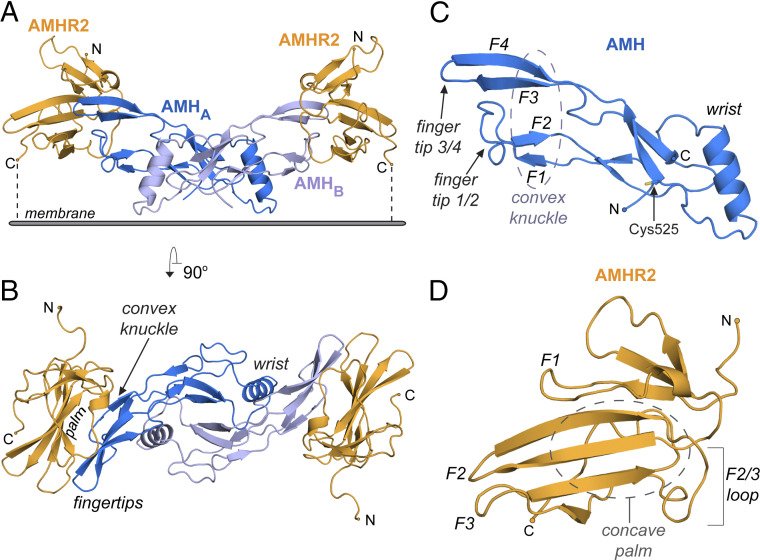
Crystal Structure of AMH (blue) bound to AMHR2 ECD (orange). (*A*) Structure of AMH bound to AMHR2 with C terminus of AMHR2 pointed toward the cell membrane. Each monomer of AMH is colored in a different shade of blue. N and C terminus of AMHR2 are labeled. (*B*) Structure of AMH bound to AMHR2 rotated 90° outward to give a top view. Features of AMH and AMHR2 are labeled, including the concave palm of AMHR2 and convex knuckle, wrist, and fingertips of AMH. (*C*) Structure of monomeric AMH with features labeled and Cys525 highlighted in yellow. (*D*) Structure of AMHR2 ECD with features labeled.

The ECD of AMHR2 residues 18 to 124 was cloned into a construct containing a cleavable maltose-binding protein (MBP) fusion and octahistidine tag for baculoviral expression in SF+ cells. Insect cell media containing the MPB-AMHR2 was subjected to Ni-affinity chromatography, size-exclusion chromatography (SEC), tag removal, and a final SEC. Excess AMHR2 was combined with the AMH ligand and purified by SEC to isolate the binary AMH/AMHR2 complex from improperly folded AMHR2. Diffraction quality crystals of the AMH/AMHR2 complex were obtained, and the structure was solved to 2.6 Å resolution by molecular replacement (Fig. 1 and *SI Appendix*, Table S1). The asymmetric unit contains one AMH monomer and one AMHR2 ECD with the biological complex being generated by crystallographic symmetry, including a disulfide bond that connects the two AMH chains. Crystal packing is primarily driven by receptor–receptor contacts. Electron density is present for residues 459 to 560 of AMH (missing 452 to 458 of the N terminus) and all residues of AMHR2 (18 to 124), with residues at the AMH/AMHR2 interface being well defined in electron density.

The structure shows that AMH adopts the typical growth factor fold observed by other TGF-β ligands (7). As seen in Fig. 1*C*, the monomer contains four parallel fingers, numbered from bottom to top F1 to F4, comprised of beta sheets, which are tethered by a perpendicular alpha helix, termed the wrist. AMH dimerization occurs through the conserved cysteine (C525) to give the disulfide-linked signaling molecule, in which the wrist helix makes up, the dimer interface, and the knuckles form a convex surface for AMHR2 binding (Fig. 1).

Fig. 1*D* shows the structure of AMHR2, which adopts the three-finger toxin fold, as observed in other TGF-β type II receptors (7). These receptor structures resemble a hand with three fingers, numbered F1 to F3 from top to bottom, converging together on one end into the palm region. Similar to the Activin and BMP type II receptors, the palm of AMHR2 creates a concave surface for ligand binding. (Fig. 1*D*).

### Structural Analysis of AMH/AMHR2 Complex.

As expected, two AMHR2 receptors are bound to the AMH ligand, in which a single receptor exclusively contacts a single chain of AMH (Fig. 1 *A* and *B*). The AMH/AMHR2 interface is significant with an interface area of 906 Å^2^. Fig. 1*A* represents the anticipated orientation of the complex position on the cell surface, with the C-terminal tails of the receptor pointing down toward the cellular membrane. AMHR2 binds to AMH using the concave surface created by the palm of the receptor, which binds to the convex knuckle surface of AMH. This results in the fingers of the receptor, namely the β-strands, being positioned parallel to the finger-like β-strands of the ligand (Fig. 1*A*). In addition to the central palm–ligand interface, AMHR2 wraps around fingertip 3/4 of AMH through extensions in both fingers 1 and 3 of AMHR2.

**Fig. 2.**
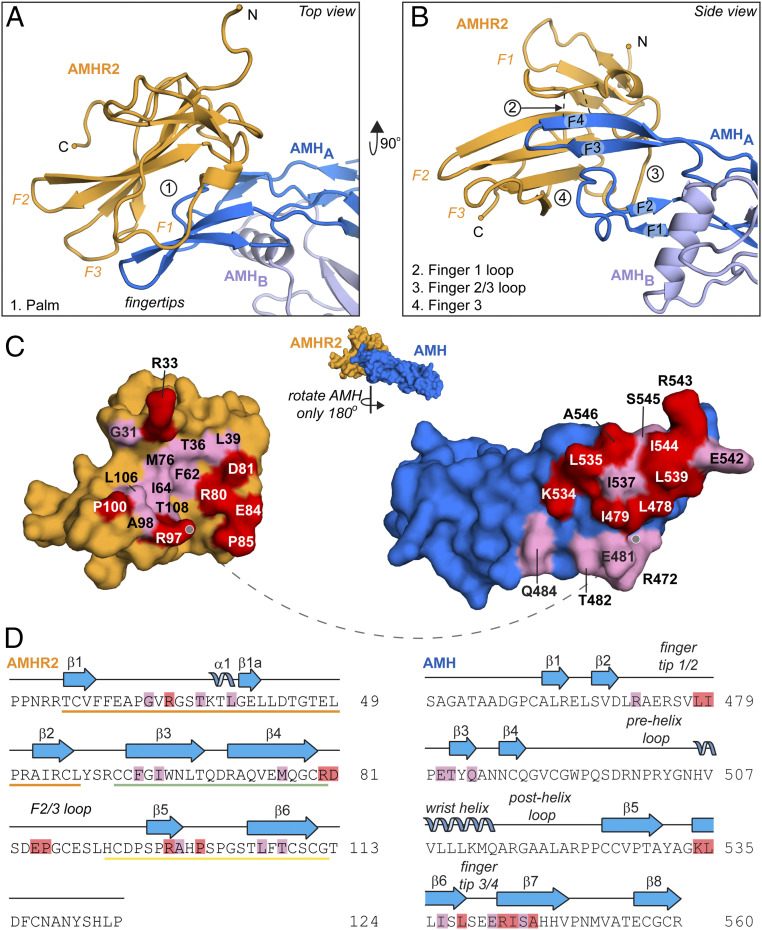
Close-up of AMH:AMHR2 interface and binding sites. Top (*A*) and side (*B*) view of AMH bound to AMHR2. Regions of contact between receptor and ligand are labeled in relation to the receptor features as the following: 1. Palm, 2. Finger 1 loop, 3. Finger 2/3 loop, and 4. Finger 3. Dotted black lines between chains indicate hydrogen bonds. (*C*) Pull apart of the ligand–receptor interface of AMH (blue) and AMHR2 (orange), rotating only AMH 180° away from AMHR2. Residues with a BSA 20 to 50 Å^2^ are highlighted in pink, and those with a BSA >50 Å^2^ are highlighted in red. Dotted line shows the anchor point. (*D*) Sequence of AMHR2 (*Left*) and AMH (*Right*), with secondary structure elements labeled. Residues are highlighted to match BSA values in *C*. Residues located in finger 1 (orange), finger 2 (green), and finger 3 (yellow) are underlined in respective colors in the sequence of AMHR2.

To further analyze the AMH/AMHR2 interface, we identified residues within the interface with a buried surface area (BSA) greater than 20 Å^2^ (21). These residues are highlighted in the surface representation of AMH and AMHR2 in Fig. 2*C* and annotated on the sequence shown at the bottom of Fig. 2. Residues with a BSA of 20 to 50 Å^2^ are highlighted in pink, while residues with a BSA >50 Å^2^ are highlighted in red. Interestingly, residues with more BSA are located peripheral to the central interface of the palm region of AMHR2, indicating that important interfacial residues extend well beyond the central core of AMHR2.

The components of AMHR2 that contact AMH can be categorized into four main contact points: 1) the palm, 2) the finger 1 loop, 3) the finger 2/3 loop, and 4) finger 3 (Fig. 2 *A* and *B*). Both the palm and finger 1 loop are centrally located, while the finger 2/3 loop and finger 3 form contacts at the peripheral edge of the interface. First, at the center of the interface, there are several hydrophobic residues within the palm of AMHR2, including F62, I64, M76 and L106, which bind to corresponding hydrophobic residues on the knuckle region of AMH (L478, I479, L535, I537, L539, I544, and A546) (Fig. 2 and *SI Appendix*, Fig. S1, box 1). Second, also centrally located, an unstructured loop within finger 1 of AMHR2 extends toward the interface and creates a short β-parallel interaction with the top of finger 4 of AMH. This positioning facilitates the formation of main-chain hydrogen bonds between residue R33 of AMHR2 and residues I544 and A546 of AMH (Fig. 2 and *SI Appendix*, Fig. S1, box 2). This main-chain hydrogen bond feature is unique to AMHR2 and has not been observed with other type II ligand interactions. Third, finger 2/3 of AMHR2 interacts with the knuckle region of AMH, where residues D81, E84, and P85 in the finger 2/3 loop of AMHR2 interact with residues Q484 and K534 of AMH. Here, a salt bridge is formed between AMHR2 residues D81 and E84 and AMH residue K534 (Fig. 2 and *SI Appendix*, Fig. S1, box 3). Lastly, finger 3 in AMHR2 interacts with the tip of the ligand through interactions with fingertip 1/2, in which R97 of AMHR2 contacts the main chain of AMH (Fig. 2 and *SI Appendix*, Fig. S1, box 4).

### Functional Analysis of the AMH/AMHR2 Molecular Interface.

**Fig. 3.**
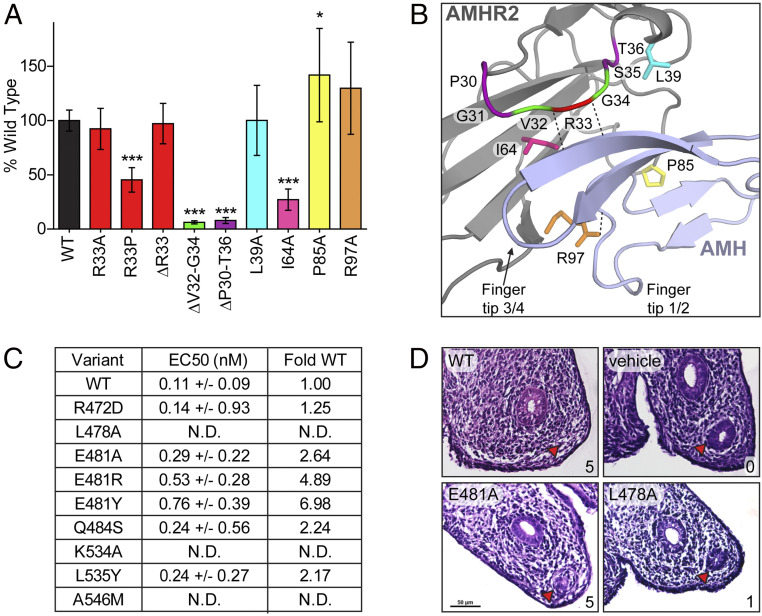
Functional analysis of the AMH- and AMHR2-binding interface. (*A*) Effects of AMHR2 point mutations or deletions (Δ) on AMH signal plotted as percent of WT signal. Δ indicates residues deleted within the finger 1 loop of AMHR2. Error bars show SD of three independent plates, with all data points performed in triplicate per plate. The colors of bars are labeled based on coloring in the structure in *D*. (*B*) Structure close up of AMH (light blue) and AMHR2 *(*gray). Residues tested in the AMHR2 luciferase assay are labeled and colored to match the graph in *C*. Dotted black lines between chains indicate hydrogen bonds. (*P ≤ 0.05 and ***P ≤ 0.001, one-way ANOVA with Bonferroni’s multiple comparison test). (*C*) Table summarizing EC_50_ of WT or mutant AMH and fold change, compared to WT. Fold change was calculated by dividing the EC_50_ of the mutant by the EC_50_ of WT AMH. N.D.= not detected. (*D*) Rat fetal UGRs treated with either WT, E481A, L478A, or vehicle control AMH. Red arrow is pointed toward the Müllerian duct. Scores from 0 (no regression) to 5 (full regression) are in the bottom right corner of each box. Scale in E481A is maintained within other ridges.

Previous studies have investigated the AMHR2 ligand-binding interface using a structural model of the binary complex (19, 20). These studies concluded that AMHR2 uses the concave palm surface for interactions with AMH; however, these limited studies were conducted prior to having a solved structure. Examination of the AMH/AMHR2 structure has revealed additional regions within AMHR2 that are involved in AMH binding. To determine the contribution of these previously unexplored regions of AMHR2 on AMH binding, we used site-directed and deletion mutagenesis to generate AMHR2 constructs with mutations in these regions. As mentioned, a unique part of the AMHR2 structure is an extension in the finger 1 loop, which uses the backbone carboxyl and amino groups of R33 to form hydrogen bonds with the backbone carboxyl group of I544 and amino group of A546 in AMH finger 4 (Fig. 2 and *SI Appendix*, Fig. S1 box 2). To determine if this interaction is important for AMH signaling, we generated two single-point mutations (R33A and R33P) and three deletions (ΔR33, ΔV32-G34, and ΔP30-T36) and examined if disruption of this interaction would impact AMH signaling. We tested how these modifications would impact AMH signaling in a cell-based (BRE-human embryonic kidney [HEK] 293) luciferase AMH reporter assay, in which wild-type (WT) or variant AMHR2 was transfected (20). The results showed that an alanine mutation or deletion of AMHR2 residue R33 exhibited normal AMH signaling. However, replacing R33 with a proline, to disrupt the main-chain hydrogen bonding interactions, showed a 50% decrease in AMH signal. Interestingly, if we deleted multiple residues of finger 1 (V32-G34 or P30-T36), we ablated AMH signaling (Fig. 3 *A* and *B*). These results were not impacted by different levels of AMHR2, as Western blot analysis showed the variants and WT receptors were expressed to similar levels; however, we cannot discount the possibility that these deletions had a negative effect on the folding or stability of the receptor (*SI Appendix*, Fig. S2). Additionally, we made AMHR2 constructs with single-point mutations of L39A, I64A, P85A, and R97A to test the impact of residues in other locations on the receptor. We found that I64A, located within the hydrophobic palm, dramatically decreased the AMH signal; however, L39A, located at the finger 1 fingertip, and R97A, located on the finger 3 fingertip, had no significant effect on signal. Furthermore, P85A, located in the finger 2/3 loop, showed a slight increase in AMH signal (Fig. 3 *A* and *B*).

In addition to investigating residues within AMHR2, we targeted residues within AMH that were likely to affect receptor binding, based on the structure of the complex. We started by generating alanine or glutamate mutations to the 10 residues with the highest BSA, as determined by jsPISA. These residues included L478, I479, E481, K534, L535, L537, L539, R543, I544, and A546. However, after generating these constructs and testing expression using an antibody against the N-terminal prodomain to avoid detection problems from mutation, we observed that the majority of mutations made to residues located within the knuckle region of AMH, especially those which are hydrophobic, either expressed poorly or did not express. As a result, we proceeded with testing the function of only the variants that were readily expressed (L478A, E481A, and K534A). These mutant AMH constructs were overexpressed in mammalian cells, purified by affinity chromatography, and tested in an AMH responsive luciferase assay. As summarized in Fig. 3*C*, L478A and K534A completely abolished AMH signal, while E481A showed only a slight decrease in AMH potency. Furthermore, we analyzed the effects of two of these AMH variants on Müllerian duct regression using a fetal rat urogenital ridge (UGR) assay. As previously described, this assay measures the direct biological function of AMH (22, 23). Regression of the Müllerian duct can be scored on a 0 to 5 scale, in which 0 is no regression and 5 is full regression of the duct. In the UGR, we were able to achieve nearly full Müllerian duct regression from E481A (score of 4 to 5), while L478A had little effect on duct regression (score of 1) (Fig. 3*D*). This data correlates with the observed activity in the AMH luciferase assay (Fig. 3*C*). In an attempt to identify mutations to residues within this interface which still expressed, we made several conserved and nonconserved mutations to residues within the knuckle region. Those which expressed (R472D, E481R, E481Y, Q484S, L535Y, and A546M) were purified by affinity chromatography and tested in the AMH luciferase assay. We found that, unlike the E481A mutant, E481R and E481Y decreased potency by ∼five to sevenfold. Additionally, A546M ablated the AMH signal. However, R472D, Q484S, and L535Y had little effect on potency (Fig. 3*C*).

In summary, our data shows that mutations to most hydrophobic residues within the knuckle of AMH resulted in a significant decrease in expression and mutation of L478, located in the knuckle, to an alanine ablated AMH signal. Moreover, mutating K534 to an alanine, thus breaking the salt bridge between K534 in AMH and D81/E84 in AMHR2, ablated AMH signaling, indicating that this interaction is essential to AMH function. Lastly, mutating A546 to a methionine also ablated AMH signal, likely due to steric clashes with finger 1 of AMHR2.

### Structural Comparison to TGF-βs, Activins, and BMPs.

Previous structural studies of TGF-β ligands and receptors have provided a good understanding for how different type II receptors bind and engage different ligands. In general, ligands adopt a very similar shape in which sequence differences paired with minor structural differences account for the specificity of ligand–type II pairings (7, 24). Now having the structures of AMH and AMHR2, we wanted to perform a cross-comparison to other ligands in the family as well as other type II TGF-β receptors. Finally, we compared the binary structure of AMH/AMHR2 to the other type II ligand complex structures.

Overall, the structure of the AMH ligand resembles other family members with an rmsd of the C-α positions of 1.78 Å for BMP2, 1.97 Å for GDF11, and 1.54 Å for TGF-β1. The core β-strands, including the cystine knot, are structurally similar, with the most significant variation occurring in the fingertips and in the wrist region. The most significant difference occurs with the shape of fingertip 3/4, which adopts a more flattened or extended conformation, similar to TGF-β ligands but starkly different from BMP and Activin ligands, which tend to curl toward the wrist helix (*SI Appendix*, Fig. S4). Interestingly, AMH lacks two conserved tryptophan residues within fingertip 1/2 present in all other ligands (*SI Appendix*, Fig. S4*E*). This causes a slight truncation of the fingertip 1/2 loop in AMH. Based on our complex structure, this truncation may be important for AMH binding to AMHR2, as a more elongated fingertip would likely clash with finger 3 of AMHR2. Furthermore, the flattened fingers and the absence of the two conserved tryptophan residues will likely impact type I receptor binding and might in part be responsible for the low affinity, as compared to the BMP ligands binding to similar receptors (i.e., Alk2, Alk3, and Alk6).

**Fig. 4.**
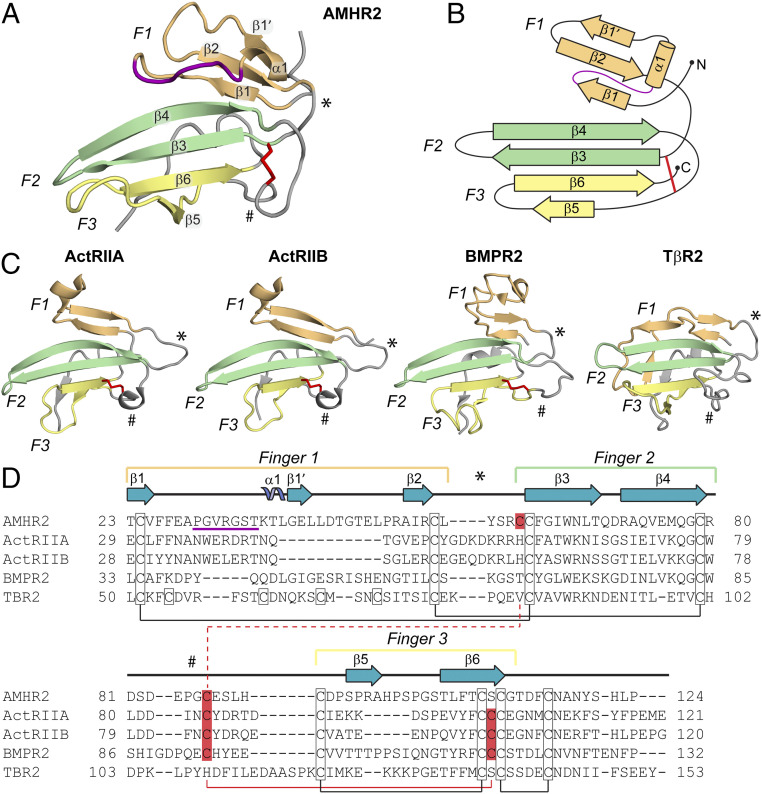
Comparison of the TGF-β family type II receptor structures. (*A*) Structure of AMHR2 ECD with finger 1 (F1, light orange), finger 2 (F2, light green), and finger 3 (F3, light yellow) labeled. Finger 1 loop in AMHR2 is colored in purple. Secondary structure elements are labeled. * indicates the finger 1/2 loop, and # indicates the finger 2/3 loop. The shuffled disulfide bond is highlighted in red sticks. (*B*) Topology map of AMHR2 with fingers colored and labeled as in *A*. (*C*) Structures of ActRIIA (PDB ID 2GOO), ActRIIB (PDB ID 2H3K), BMPR2 (PDB ID 2HLQ), and TβR2 (PDB ID 1M9Z) ECD with fingers and features labeled as in *A*. (*D*) Sequence alignment of type II receptors. Fingers 1 to 3 and loops are indicated by the brackets or symbols above the sequence colored and labeled as in *A*. Secondary structural features are labeled above the sequence accord to the AMHR2 ECD structure. Cysteines are outlined with a black box, and conserved disulfide pairs are indicated by black solid lines. The nonconserved disulfide is highlighted in red. The solid red line shows the disulfide pair in ActRIIA/ActRIIB/BMPR2, while the dotted red line shows the disulfide pair in AMHR2. Residue numbers are labeled at the beginning and end of each line.

With AMHR2 being the final type II structure to be solved of the family, we have an opportunity to compare AMHR2 to the other type II receptors. As mentioned, type II receptors within the TGF-β family all share a similar three-finger toxin fold. However, previous structures have shown there can be differences in the length and positioning of fingers, as well as the loops connecting fingers (9). Fig. 4 depicts a comparison of AMHR2 to the other type II receptors, with a topology diagram in Fig. 4*B*, and a corresponding sequence alignment of the receptors in Fig. 4*D* (for clarity, each finger is colored separately). When superimposing the structure of AMHR2 to the four other type II receptors, AMHR2 is most similar to ActRIIB (rmsd: 1.40 Å), followed by ActRIIA (rmsd: 1.46 Å), Tβ-R2 (rmsd: 2.01 Å), and finally BMPR2 (rmsd: 2.15 Å).

The most significant differences across all the receptors occurs in finger 1, with additional differences in finger 3. Of all the receptors, finger 1 of AMHR2 has the most structural similarity to BMPR2, albeit modest, and adopts a significantly different structure than the other receptors. Here, finger 1 has an extension which forms important interactions with AMH, as shown by the deletion studies in Fig. 3. This feature is lacking in both ActRIIA and ActRIIB, which have a much shorter finger 1 (Fig. 4). However, ActRIIA and ActRIIB have an extension in the loop following finger 1 not observed in the AMHR2 or BMPR2, which forms an interaction with BMP and Activin ligands (see * in Fig. 4 and *SI Appendix*, Fig. S4). Following this extension, AMHR2 contains an additional beta sheet (β1′), only present in Tβ-R2 and BMPR2, that tethers finger 1 through an anti-parallel β-strand interaction with β2 (Fig. 4 *A*–*C*). Tβ-R2 also has an extension in finger 1 and a similarly located β-strand; however, this structure bends the finger in the opposite direction because of the binding of a β-strand on the C-terminal tail of Tβ-R2, which is not present in AMHR2. Furthermore, Tβ-R2 uses its unique fold of finger 1 as its main interface to bind its cognate ligands, TGF-β1 to TGF-β3 (10, 25–27). Thus, across the type II receptors, finger 1 has evolved different conformations that are important for ligand binding and specificity (Fig. 4).

Type II receptors in the TGF-β family are highly disulfide bonded. In general, four disulfide bonds are conserved between type II receptors, which are located on the opposite side of the palm, facing away from the ligand interface. Excluding Tβ-R2, the type II receptors have an additional disulfide bond positioned near the ligand interface. Interestingly, the position of this disulfide bond is altered within AMHR2 and represents a unique aspect of the structure of AMHR2. For ActRIIA, ActRIIB, and BMPR2, this disulfide bond links the finger 2/3 loop to β6 of finger 3, while for AMHR2, there is a shift in the placement of one of the cysteines. The cysteine normally positioned in β6 is shifted to β3 of finger 2 (Cys60), while the bonding cysteine remains in the finger 2/3 loop. As a result, the shifted disulfide bond in AMHR2 tethers the finger 2/3 loop to finger 2, (Fig. 4 *A* and *D*). This unique disulfide bond appears to be important for AMH binding since the finger 2/3 loop interacts with the knuckle region of AMH.

**Fig. 5.**
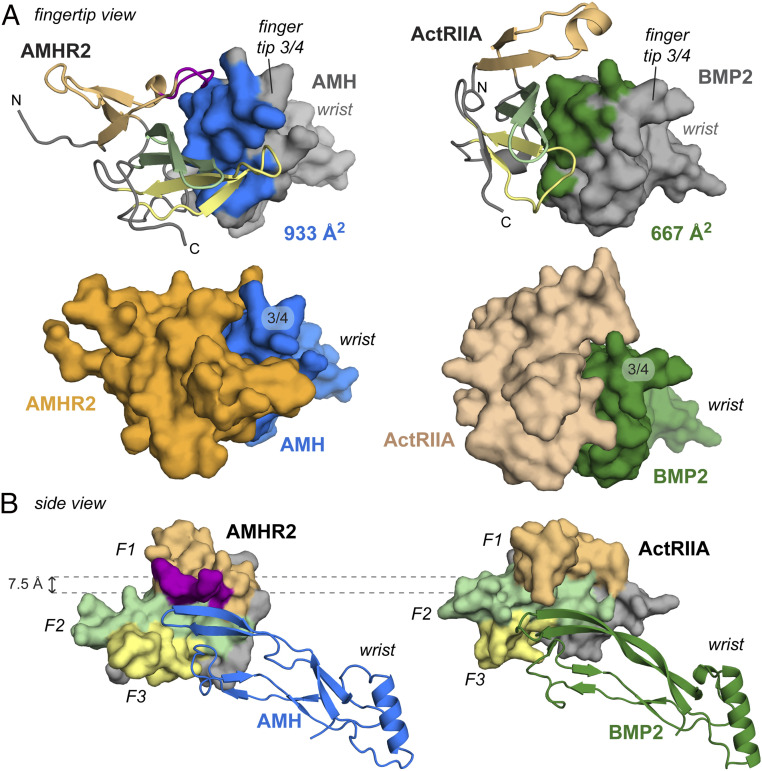
Comparison of type II receptor binding between AMH and BMP2. (*A*) Fingertip view of AMH bound to AMHR2 (*Left*) and BMP2 bound to ActRIIA (*Right*, PDB ID 2GOO). Fingertip 3/4 and wrist are labeled. Upper panels show the surface area usage within the ligand highlighted in blue (AMH) or green (BMP2). Receptors are colored according to previous figures with finger 1 in light orange, finger 2 in light green, and finger 3 in light yellow. The finger 1 loop in AMHR2 is colored in purple. Bottom panels show both receptor (AMHR2, orange and ActRIIA, wheat) and ligand (AMH, blue and BMP2, green) in surface representation. (*B*) Side view of AMH:AMHR2 binary (*Left*) and BMP2:ActRIIA binary (*Right*). Fingers of the receptor are labeled F1 to F3 and colored according to *A*. AMHR2 is positioned 7.5 Å lower on AMH than ActRIIA is on BMP2.

**Fig. 6.**
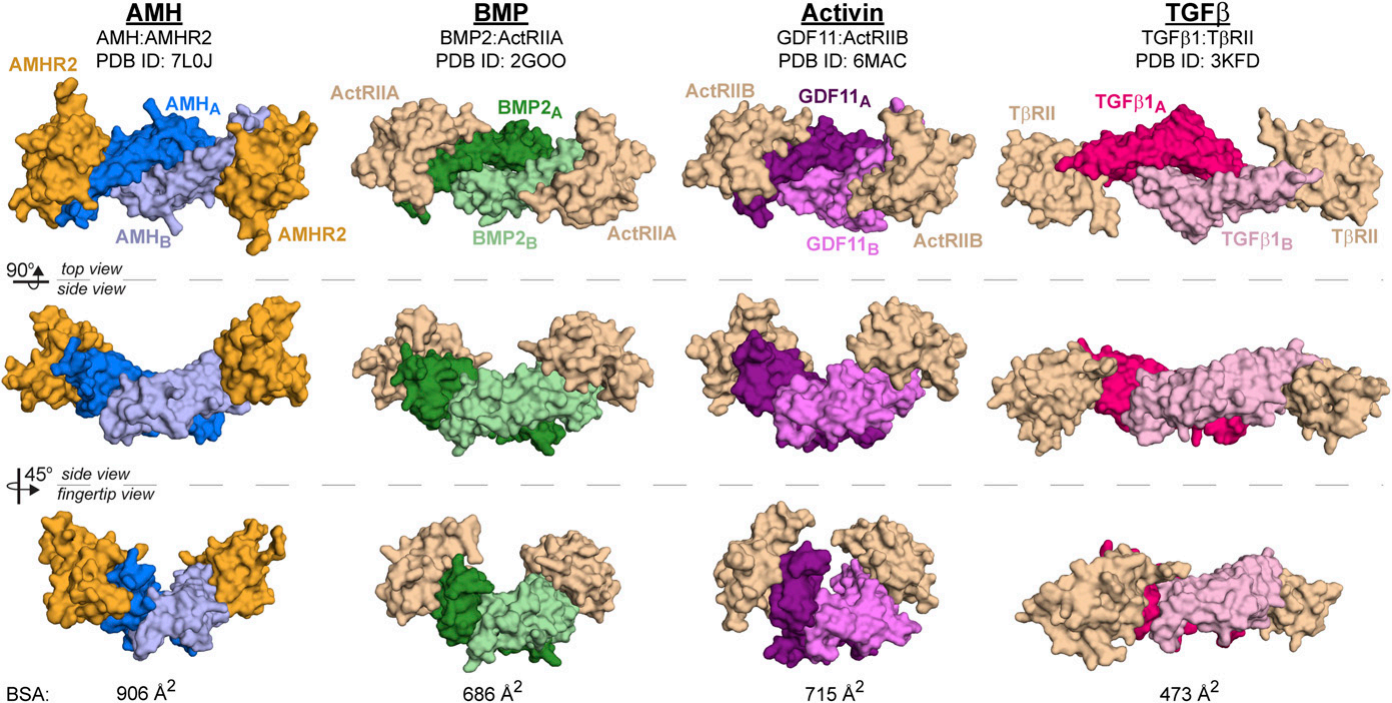
Comparison of the binary complex structures of each TGF-β class. Top view (*Top*), side view (*Middle*), and fingertip view (*Bottom*) of AMH (blue), BMP (green), Activin (purple), and TGF-β (pink) class ligands in complex with their type II receptors. AMHR2 is colored in orange, and all other type II receptors are colored in wheat. BSA at the ligand/receptor interface is shown at the bottom of each column.

Previous structural studies of TGF-β family complexes have revealed two mechanisms for type II receptor binding. Activins and BMPs bind their type II receptor using the knuckle region of the ligand to bind the palm of the receptor. Alternatively, TGF-β ligands bind their type II receptor using the fingertips of the ligand to bind finger 1 of the receptor (7). The structure of AMH bound to AMHR2 reveals that AMHR2 binds most similar to how Activin and BMP ligands bind their type II receptors and less like the TGF-β complex. Like Activins and BMPs, AMHR2 uses the concave palm region to bind to the convex knuckle region of AMH. Despite this similarity, there are differences in the placement of AMHR2, when compared to that of ActRIIA and ActRIIB, highlighted through the comparison in Fig. 5. One striking difference is a shift in the position of the receptor relative to the ligand (Fig. 5*B*). Comparison shows that the receptors are shifted by about 7.5 Å, where the AMHR2 position is shifted toward finger 1/2 of the ligand, while ActRIIA and ActRIIB bind Activins and BMPs toward the top of finger 4. To highlight these positional differences, the footprint of both AMHR2 binding to AMH and ActRIIA binding to BMP2 are shown in the supplement (*SI Appendix*, Fig. S4). As shown in *SI Appendix*, Fig. S5*A*, AMHR2 more readily cups the ligand through interactions on both the top and bottom of the ligand. As a result, more surface area on AMH is covered by AMHR2 (933 Å^2^), compared to the surface area on BMP2 covered by ActRIIA (667 Å^2^) (Fig. 5*A*). Furthermore, the AMH and AMHR2 interface area is more extensive than the other type II receptors [BMPs Protein Data Bank (PDB) ID 2GOO (12): 686 Å^2^, Activins PDB ID 6MAC (13): 715 Å^2^, and TGF-βs PDB ID 3KFD (26): 473 Å^2^] (Figs. 5 and 6). While the BSA does not necessarily predict binding affinity, the unique interactions afforded by AMHR2 drive the observed ligand specificity. Thus, while AMHR2 binds in a mode similar to how type II receptors engage BMP and Activin ligands, the structure reveals that AMHR2 has evolved significant modifications that permit exclusive binding to the AMH ligand.

## Discussion

AMH plays an important role in several reproductive functions, including Müllerian duct regression and regulation of folliculogenesis. As a result, AMH has been implicated in several reproductive disorders and cancers and is thus a target of interest in therapeutic intervention of these diseases and as a contraceptive agent also capable of protecting ovarian reserve during chemotherapy and enhancing yield during in vitro fertilization (4, 6). Furthermore, because AMH is the only ligand which binds AMHR2, this makes AMH and AMHR2 ideal for therapeutic targeting, while limiting potential off target effects. However, the lack of experimental structural information makes rational design of drug therapies difficult. While efforts have been made to model the structure of AMH bound to AMHR2, the exact fold of the receptor- and ligand-binding interface remained unclear (19, 20, 28, 29). Furthermore, some of these studies have used these models in an attempt to characterize antibodies designed to bind AMHR2, specifically for use in targeted cancer cells expressing high levels of AMHR2 (19, 28). Interestingly, all models of AMHR2 were inaccurate and unable to predict the fold of the finger 1 loop extension in the receptor. Furthermore, these models failed to predict the altered positioning of AMHR2 on AMH. As such, this study provides much needed experimental evidence for characterizing, at the molecular level, how AMH specifically binds and signals through AMHR2.

Because mutations in AMH and AMHR2 have been identified in patients with PMDS (3), we had the opportunity to use the structure to understand how these mutations may affect AMH signaling through AMHR2. Two PMDS mutations that have been identified in AMHR2, M76V and D81E, map to the AMH/AMHR2 interface (*SI Appendix*, Fig. S3), which we previously tested and showed these mutations had no significant effect on AMH signaling in a cell-based luciferase reporter assay (20). While clearly at the interface of the ligand, these mutations are relatively conserved, which indicates that Müllerian duct regression activity might be sensitive to subtle changes at this interface and suggests that signaling must be tightly regulated. Interestingly, the majority of PMDS mutations are located outside of the ligand/type II receptor interface, suggesting that they are likely disrupting the folding of these proteins (3). For example, a mutation of R59C in the receptor results in a loss of a hydrogen bond in the receptor and also the addition of a free cysteine, which could interfere with the proper disulfide bond architecture. Additionally, a mutation in AMH (C525Y) disrupts the intermolecular disulfide bond and likely disrupts dimerization and receptor assembly. Curiously, mutations within AMH appear to be also located in the putative type I receptor-binding interface, including mutations in the prehelix loop (Q496H), the wrist helix (H506Q), and the concave surface of finger 2 of AMH (V477A).

Structural studies of TGF-β family member complexes have been integral in understanding how ligand–receptor specificity occurs within this large protein family. In general, structures of ligands in complex with their type II receptors have shown two distinct binding mechanisms: the Activins and BMPs, which use the knuckle of the ligand to bind the palm of the receptor, and the TGF-βs, which use the fingertips of the ligand to bind finger 1 of the receptor (Fig. 6). While AMH shares characteristics with the Activin and BMP classes and might bind in a similar location, it was unclear exactly how AMH interacted with AMHR2 to maintain specificity (19, 20). In other words, if there is a common binding location for the type II receptors, why then are Activin and BMP ligands unable to bind to AMHR2, and vice versa? Also, why are other ligands unable to utilize AMHR2 as a type II receptor? Additionally, given that AMH and AMHR2 only share ∼20% sequence similarity to their closest homolog (*SI Appendix*, Table S2), we wondered how the structures of these molecules would compare to other TGF-β ligands and type II receptors. The structure of AMH bound to AMHR2 now allows us to start answering these questions. While overall binding is similar in location to the BMP and Activin ligands, unlike other ligand–type II receptor complexes, the structure of AMH bound to AMHR2 shows that the receptor more extensively wraps around the ligand, creating unique ligand–receptor contact points (Fig. 6). This is created through several of the previously mentioned structural features in AMHR2, which are not conserved between type II receptors, primarily the finger 1 loop and finger 3 of AMHR2, and residues in AMH, which are not conserved in other ligands (*SI Appendix*, Fig. S5).

One primary difference, shown in Fig. 4, between AMHR2 and the type II receptors used by Activins and BMPs is an extension in finger 1. As mentioned, BMPR2 also has an extension in finger 1, compared to ActRIIA and ActRIIB, though not as long as the one in AMHR2. As shown by the purple underline in the sequence alignment in Fig. 4, AMHR2 has an additional five residues in finger 1 not present in BMPR2. Importantly, the structure of AMHR2 bound to AMH shows that these residues create a unique ligand-binding site, which when deleted, abolished AMH signal (Fig. 3). The unique fold and extension in AMHR2 create a flat loop, which sits parallel to finger 4 of AMH, capping the top of the ligand (Figs. 4 and 5).

One possible reason that the interaction with the finger 1 loop of AMHR2 is AMH specific is the positioning of an alanine (A546), located on finger 4 of AMH. The short sidechain of the alanine allows the finger 1 loop of AMHR2 to position close enough to AMH to bind through main-chain hydrogen bonds, without clashing with the sidechain of residues on AMH. In fact, a sequence alignment of AMH with the other ligands within the TGF-β family shows a much larger sidechain positioned in this location in all other ligands, which would likely cause clashes between the finger 1 loop of AMHR2 and finger 4 of the ligand. (*SI Appendix*, Fig. S5). Furthermore, when we replaced this residue with a methionine, we ablated AMH signal. As such, the larger sidechains found in other ligands would likely create steric clashes with AMHR2, thus inhibiting binding of other ligands to AMHR2.

In addition to the unique fold of finger 1 of AMHR2, the lower positioning of the receptor and shuffled disulfide bond places the AMHR2 finger 2/3 loop closer to AMH, forming contacts with finger 2 of AMH (*SI Appendix*, Fig. S1, box 3). As mentioned, this contact forms salt bridges between residues D81 and E84 of AMHR2 and K534 of AMH, which are not present in other ligand type II receptor pairs (*SI Appendix*, Fig. S5). Furthermore, the sidechain of E84 in AMHR2 projects into the ligand/receptor interface. As a result, finger 2 in both BMP and Activin ligands, which is more extended, would clash with this residue, alluding to another reason for the observed specificity between AMH and AMRH2. Lastly, the extended finger 3 of AMHR2 forms contacts with fingertip 1 of AMH, a contact not observed with the type II interactions with BMPs or Activins (Fig. 2 and *SI Appendix*, Fig. S1). For BMPs and Activins, this fingertip is extended by 2 to 3 residues and would lead to a steric clash with AMHR2 (Fig. 5 and *SI Appendix*, Fig. S5). Collectively, these exclusive binding interactions and the unique fold of AMHR2 begin to explain why AMH is specific to AMHR2.

Aside from the unique features in AMHR2 responsible for specificity, AMH also has several features which inhibit binding to other type II receptors. In general, Activin and BMP type II receptors utilize a hydrophobic, aromatic triad (residues Tyr, Trp, and Phe) to bind corresponding hydrophobic residues (Ile, Ala, and Pro) on the ligand. If we were to superimpose AMH to GDF11, in the structure with ActRIIB (PDB ID: 6MAC), differences in the AMH ligand show that similar hydrophobic core interactions would not occur. For instance, L535 in AMH is positioned in such a way that it would likely clash with the tryptophan of the triad found in Activin and BMP type II receptors, while in Activin and BMP ligands, this residue is a small polar residue. Alternatively, the structure also reveals why other type II receptors will not bind AMH. For example, superposition of the Activin and BMP type II receptors onto AMHR2 shows that I479 of AMH would sterically clash with the triad of hydrophobic residues within the core of the interface of ActRIIA/ActRIIB/BMPR2. For ligands that bind ActRIIA/ActRIIB/BMPR2, the I479 is replaced with an alanine. Interestingly, this is modified in other TGF-β ligands to avoid type II receptor binding (e.g., a glutamate in TGF-β ligands). Therefore, interactions between the Activin and BMP type II receptors and AMH are unlikely to occur because of these amino acid differences.

In addition to understanding how AMH maintains specificity for its type II receptor, AMHR2, it is interesting to postulate how these differences may affect type I receptor binding. As mentioned, BMP ligands bind their type I receptors with high affinity, while Activin and TGF-β ligands bind type I receptors with low affinity (7). From structures of different ligand classes in complex with type I receptors, it has been shown that three paradigms exist for binding type I receptors (7). The BMPs are rigid and use a lock-and-key mechanism to readily bind type I receptors with high affinity (12). Alternatively, both Activin and TGF-β class ligands have developed compensation techniques for binding to their low-affinity type I receptors. The TGF-β ligands use a cooperative mechanism, in which the high-affinity type II receptor creates contacts with the type I receptor (10). Alternatively, the Activin ligands use a conformational selection mechanism, in which contacts with both the fingertips and wrist helix of the ligand accommodate type I receptor binding (13). Studies have shown that AMH signals through the type I receptors used by BMPs (Alk 2, Alk3, and Alk6) (30–33). However, unlike BMPs, it has been suggested that AMH likely has low affinity for its type I receptors, similar to Activins (34). Interestingly, the structural differences in AMH suggest that AMH will bind these type I receptors significantly differently. For example, a common knob-in-hole mechanism is used by Activin and BMP ligands, in which a conserved phenylalanine in the type I receptor inserts between two conserved tryptophan residues on fingertip 1/2 of the ligand (12, 13). However, these tryptophan residues are absent in AMH, implying that type I receptor binding is likely altered (*SI Appendix*, Fig. S5*E*). Moreover, AMH does not have the curved fingertip 3/4 used by Activin ligands to bind type I receptors (*SI Appendix*, Fig. S5). As such, these structural differences in AMH suggest that AMH will bind type I receptors significantly different from BMPs or Activins. Ultimately, a ternary structure of AMH/AMHR2/type I receptor is needed to fully understand how AMH binds a low-affinity type I receptor in the absence of mechanisms used by other TGF-β family ligands.

In conclusion, the structure of AMH bound to AMHR2 shows that this unique pair has evolved changes in both the ligand and receptor to maintain specificity. Overall, the structure shows a modified Activin/BMP type II receptor-binding mode, in which AMHR2 is shifted downward and wraps around fingers 3 and 4 of AMH (Fig. 6). Furthermore, AMHR2 uses the main chain of finger 1 to hydrogen bond with the main chain of finger 4 in AMH, resulting in the only ligand/type II receptor pair to bind in this manner. In summary, the structure of AMH bound to AMHR2 provides valuable insight into how the two proteins engage, which can be used for the rational design of therapies targeting the AMH/AMHR2 interface to prevent or enhance signaling.

## Materials and Methods

### Expression and Purification of AMH for Structural Studies.

Recombinant AMH protein was expressed and purified, as previously reported (6, 20). Briefly, AMH with an enhanced cleavage site albumin leader, Q425R MIS (LR-MIS) was produced using a stable Chinese hamster ovary cell line (6, 23). Conditioned media was purified using an affinity chromatography column, with 6E11 antibody primary amino coupled to an N-hydroxysuccinimide (NHS) column (Cytiva) (6, 23, 35). AMH mature was then purified away from the prodomain by reverse phase chromatography and stored for future use, as previously reported (20).

### Expression and Purification of AMHR2 ECD for Structural Studies.

AMHR2 ECD residues 18 to 124 was subcloned into the pFastBac baculovirus vector containing an N-terminal octahistidine tag, myc tag, and MBP fusion. Recombinant baculovirus was produced and amplified using the Bac-to-Bac expression system, per manufacture protocol (Invitrogen). Protein expression was conducted using standard manufacture protocols in SF+ insect cells (Protein Sciences). Cells were harvested 60 h postinfection, and cell debris was cleared through centrifugation at 3,200 rpm for 20 min at 4 °C. Conditioned media was applied to Ni Sepharose affinity resin (Cytiva) equilibrated with 20 mM Phosphate buffer pH 7.4, 500 mM NaCl, and 20 mM imidazole. Protein was eluted with equilibration buffer + 500 mM imidazole. The elutions were concentrated and applied to a HiLoad Superdex S200 16/60 column (Cytiva) in 20 mM Hepes pH 7.5 and 500 mM NaCl. Fractions containing pure AMHR2-MBP fusion were digested at 4 °C overnight with HRV-3C protease in 25 mM Tris pH 7.6, 150 mM NaCl, 1 mM EDTA, and 1% ethylene glycol. After digestions, the protein was applied to a HiLoad Superdex S75 16/60 column (Cytiva) in 20 mM Hepes pH 7.5 and 500 mM NaCl. Fractions containing pure AMHR2 ECD were dialyzed into 10 mM HCl then concentrated and stored at −80 °C until used in experiments.

### AMH:AMHR2 Complex Formation.

Purified recombinant AMH and recombinant AMHR2 ECD were mix at a 1:2.1 ratio, with receptor in excess, in 10 mM HCl and incubated at 4 °C for at least 1 h. After incubation, 500 mM MES pH 5.5 was added to a final concentration of 100 mM MES. The mixture was filtered through 0.22-μM polyethersulfone filter and loaded onto a Superdex S75 10/300 GL column (Cytiva) in 100 mM MES pH 5.5 and 150 mM NaCl. Peaks containing AMH:AMHR2 complex, confirmed by SDS-polyacrylamide gel electrophoresis, were pooled and concentrated to a final concentration of 8 mg/mL

### Crystallization and Structure Determination.

Initial crystals of the AMH:AMHR2 complex were grown in 100 mM MES pH 6.5, 200 mM lithium sulfate, and 20% polyethylene glycol (PEG) 3,350 at 20 °C by hanging drop method. These crystals, which had a needle-like morphology, were optimized to give a rectangular prism morphology. The new crystals were grown using a seed stock from the initial hit and fresh complex at 3 mg/mL in 100 mM MES pH 6.5, 200 mM lithium sulfate, 17.5% PEG 3,350, and 5% 1-butyl-3-methylimidazolium trifluoroacetate at 20 °C. Crystals were harvested and cryoprotected in mother liquor with 30% glucose. Diffraction data were collected at Argonne National Laboratories on the Advanced Photon Source GM-CA 23ID-D beamline. DIALS was used to index and integrate the data, then scaled and merged using AIMLESS (36, 37). A molecular replacement solution was identified using Phaser within the Phenix suite (38). First, GDF5 (PDB ID 1WAQ) and ActRIIB (PDB ID 6MAC) were used to generate AMH and AMHR2 models using SWISS-MODEL (13, 39, 40). The SWISS-MODEL solutions were then used as search models. Phenix.refine and Coot were used for final refinement and model building (41, 42). Coordinates were deposited to the PDB under PDB ID 7L0J (43). Interface areas and BSAs were calculated using jsPISA (21).

### Expression and Purification of AMH Mutants for Luciferase Assays.

AMH variants were cloned by site-directed mutagenesis into a full-length AMH construct (LR-MIS) (23). Constructs transiently transfection into Expi-293T cells (Life Technologies), and expression was tested by Western blot using an antibody against the prodomain (Aviva Systems Biology catalog # OAAB06540), which was not affected by mutation. Mutants which expressed well were purified by affinity chromatography using the 6E11 antibody (which recognizes an AMH epitope in a location outside of the mutated regions) and tested in the AMH luciferase assay or UGR assay for function.

### Luciferase Assays of AMH Mutations.

HEK-293T cells (1.5 × 10^4^ cells/well) were plated in poly-D-lysine (Sigma-Aldrich) coated 96-well plates (Thermo Fisher Scientific), in Dulbecco’s modified Eagle’s medium (DMEM) supplemented with 10% fetal calf serum (Life Technologies), and incubated at 37 °C in 5% CO_2_. After overnight incubation, Lipofectamine 2000 (Life Technologies) was used according to the manufacturer’s instructions to transfect cells with 100 ng/well of plasmid DNA, consisting of the following: 4xBRE-luc (98.9 ng), AMHR2 (0.8 ng) (GenScript HK Limited), and ALK2 (0.3 ng), diluted in Opti-MEM (Life Technologies). Approximately 24 h following transfection, cells were treated with increasing doses of AMH variants, diluted in serum-free medium (DMEM high glucose supplemented with 1 mM sodium pyruvate [Life Technologies] and 0.01% BSA [Sigma-Aldrich]), and incubated overnight (∼18 h) at 37 °C in 5% CO_2_. The medium was then removed, and cells were lysed in solubilization buffer (26 mM glycylglycine [pH 7.8], 16 mM MgSO_4_, 4 mM EGTA, 900 µM dithiothreitol, 1% Triton X-100). The lysate was transferred to a white 96-well plate (Corning Costar) and luminescence measured immediately after the addition of the substrate luciferin (Promega) using a CLARIOstar microplate reader (BMG Labtech).

### Luciferase Assays of AMHR2 Mutations.

Testing of AMHR2 mutations was conducted, as previously reported, using HEK-293T cells (RRID: CVCL_0063) (20, 44) Briefly, cells were plated at 20,000 cells per well and grown for 24 h. Thereafter, cells were transfected with BRE (10 ng), Alk2 (10 ng), either WT or variant AMHR2 (25 ng), and pcDNA3 empty vector (55 ng) DNA for a total of 100 ng DNA per well. At 24 h posttransfection, media was replaced with serum-free medium or with medium containing 1 nM AMH mature. At 18 h post media swap, cells were lysed with 20 μL 1× passive lysis buffer (Promega) on a plate shaker (900 rpm, 20 min, 20 °C) then transferred to a black and white 96-well plate. Then, 40 μL Luciferase Assay Reagent (Promega) was added to each well, and firefly luciferase luminescence was measured using the Synergy H1 Hybrid Plate Reader (BioTek). Each AMHR2 mutant assay was measured in at least three separate experiments and triplicated per plate. GraphPad Prism version 5 software was used to plot data and determine statistical significance by one-way ANOVA with Bonferroni’s multiple comparison test. Expression of full-length AMHR2 mutants was tested, as previously reported (20).

### Testing of AMH Mutants in UGR Assay.

The embryonic Müllerian duct regression bioassay was performed, as previously described (22, 23). Briefly, UGRs were dissected from female rat embryos at embryonic day 14.5 (E14.5). Single ridges were incubated in media containing recombinant AMH at 5 μg/mL. The contralateral ridges were treated with an equal volume of vehicle control. The ridges were cultured at the air–media interface on an agarose substrate suspended in media and incubated at 37 °C with 5% CO_2_ for 72 h. Following fixing and embedding, the tissue was sectioned, and slides were stained with hematoxylin and eosin and graded by two independent, experienced, and blinded observers on a scale of 0 to 5, with 0 representing no regression and 5 full regression of the Müllerian duct.

## Supplementary Material

Supplementary File

## Data Availability

X-ray diffraction data have been deposited in PDB (PDB ID 7L0J). All other study data are included in the article and/or supporting information.
