# Supplementary material for: Structure of AMH bound to AMHR2 provides insight into a unique signaling pair in the TGF-β family
Source: Proc Natl Acad Sci U S A. 2021 Jun 21;118(26):e2104809118. doi: 10.1073/pnas.2104809118 (PMC8256043; doi:10.1073/pnas.2104809118)
Supplement: Supplementary File [file pnas.2104809118.sapp.pdf]

Supplemental Information (SI)

Table S1. Data collection and refinement statistics of AMH:AMHR2 binary structure.

	AMH:AMHR2[a]
**Data Collection**	
Resolution range	45.68 - 2.60 (2.69 - 2.60)
Wavelength (Å)	1.03
Total reflections	57,201 (5,838)
Unique reflections	9,187 (899)
Space group	$P\ 4_1\ 2_1\ 2$
Unit Cell Dimension	
a, b, c (Å)	66.4, 66.4, 125.9
α, β, γ (°)	90, 90, 90
R_{merge}	0.098 (1.00)
R_{pim}	0.042 (0.41)
Mn (I/σI) [b]	10.12 (1.44)
$CC_{1/2}$ [c]	0.997 (0.71)
Completeness (%)	99.83 (99.89)
Multiplicity	6.20 (6.50)
**Refinement**	
Reflections used in refinement	9184 (898)
Reflections used for R-free	444 (45)
R_{work}, R_{free} [d]	0.241, 0.268
Number of non-hydrogen atoms	1628
Macromolecules	1609
Ligands	19
Protein residues	211
Root mean square deviations [e]	
Bonds (Å)	0.00
Angles (°)	0.56
Average B-factor ($Å^2$)	70.7
Macromolecules	70.4
Ligands	90.6
Wilson B-factor ($Å^2$)	64.5
Ramachandran plot [e]	
Favored (%)	97.1
Allowed (%)	2.90
Outliers (%)	0.00
Rotamer outliers (%)[e]	0.00
Clashscore [e]	2.20

[a] Values in parentheses are for highest resolution shell.
[b] Mn(I/σI) is defined as <merged<Ih>/sd(<Ih>)> ≈ signal/noise.
[c] $CC_{1/2}$ for highest resolution shell.
[d] R_{free} calculated from 5% of initial total number of reflections.
[e] Determined by MolProbity.

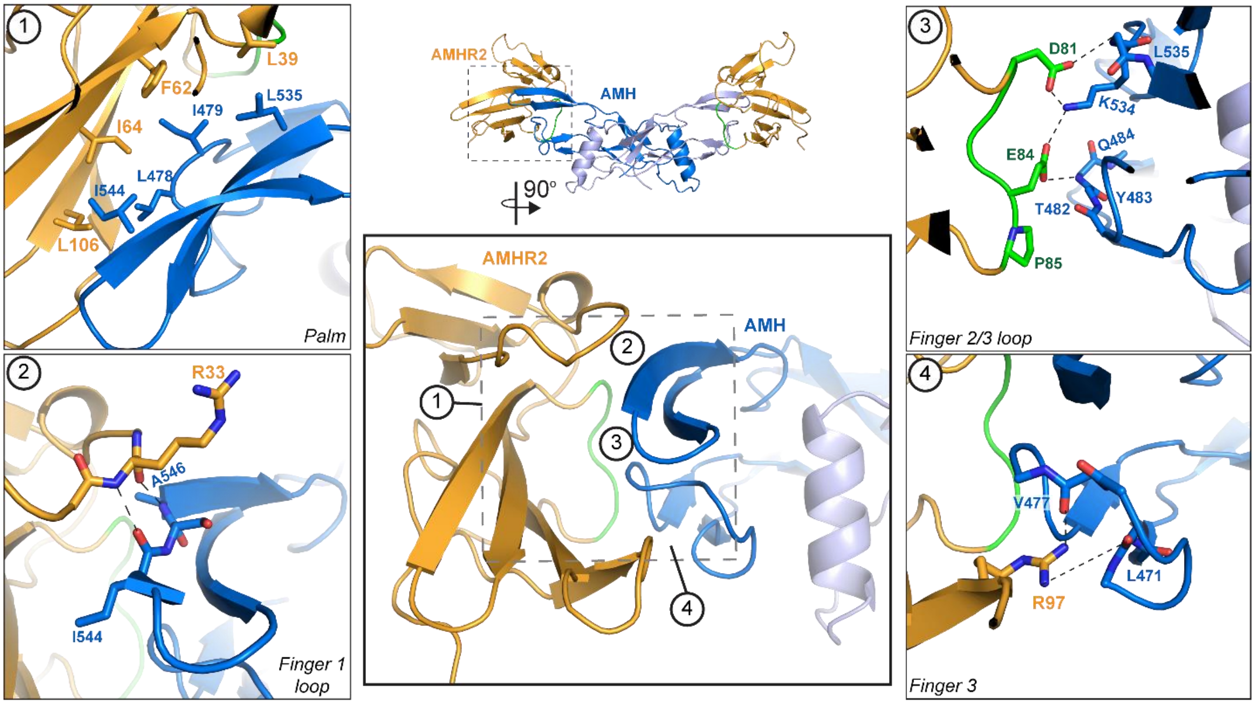

Figure S1. Residues Involved in AMH:AMHR2 Binding. Middle top shows AMH (*blue*) and AMHR2 (*orange*) structure with dotted black box indicating region zoomed in on and rotated 90° located below. Numbers show 1) palm, 2) finger 1 loop, 3) finger 2/3 loop (*green*), and 4) finger 3. Residues involved in ligand-receptor interaction are shown as sticks and labeled. Dotted black lines between chains indicate hydrogen bonds.

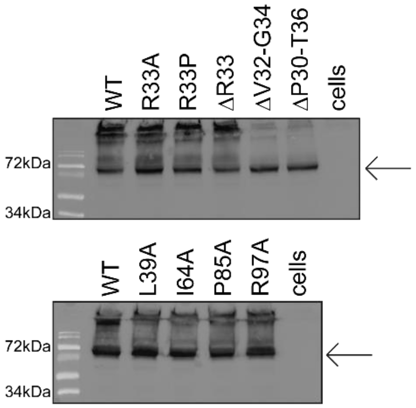

Figure S2. Western Blots of AMHR2 Mutants tested in luciferase assay in figure 3.

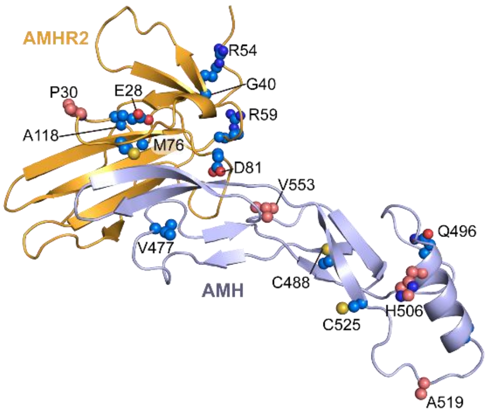

Figure S3. Residues mutated in PMDS (*blue*) and PCOS (*pink*) within the structure of AMH (*light blue*) bound to AMHR2 (*orange*). Oxygen atoms are colored in *red*, nitrogen atoms are colored in *dark blue*, and sulfur atoms are colored in *yellow*.

Table S2. Percent Identity between the Type II Receptor ECDs.

	AMHR2	ActRIIA	ActRIIB	BMPR2	TBR2
**AMHR2**	100	16.9	18.4	20	16.1
**ActRIIA**		100	53.8	19.9	14.1
**ActRIIB**			100	24.3	14.3
**BMPR2**				100	16.4
**TBR2**					100

Percent of sequence identity of the extracellular domain of each type II receptor was calculated using Clustal Omega(45).

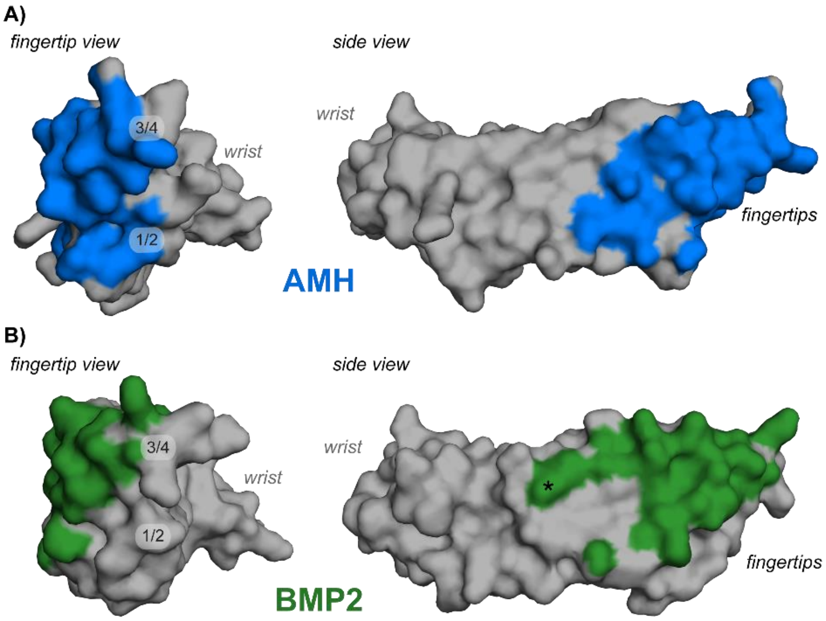

Figure S4. Receptor binding interface on AMH and BMP2. Structure of ligand monomers for AMH **(A)** and BMP2 (BMP2-PDB ID 2GOO) **(B)** from a fingertip view (*left*) and side view (*right*). All surfaces on AMH (*blue*) and BMP2 (*green*) within 5 Å of their respective type II receptors are highlighted. * in BMP2 indicates where the finger 1/2 loop of ActRIIA binds.

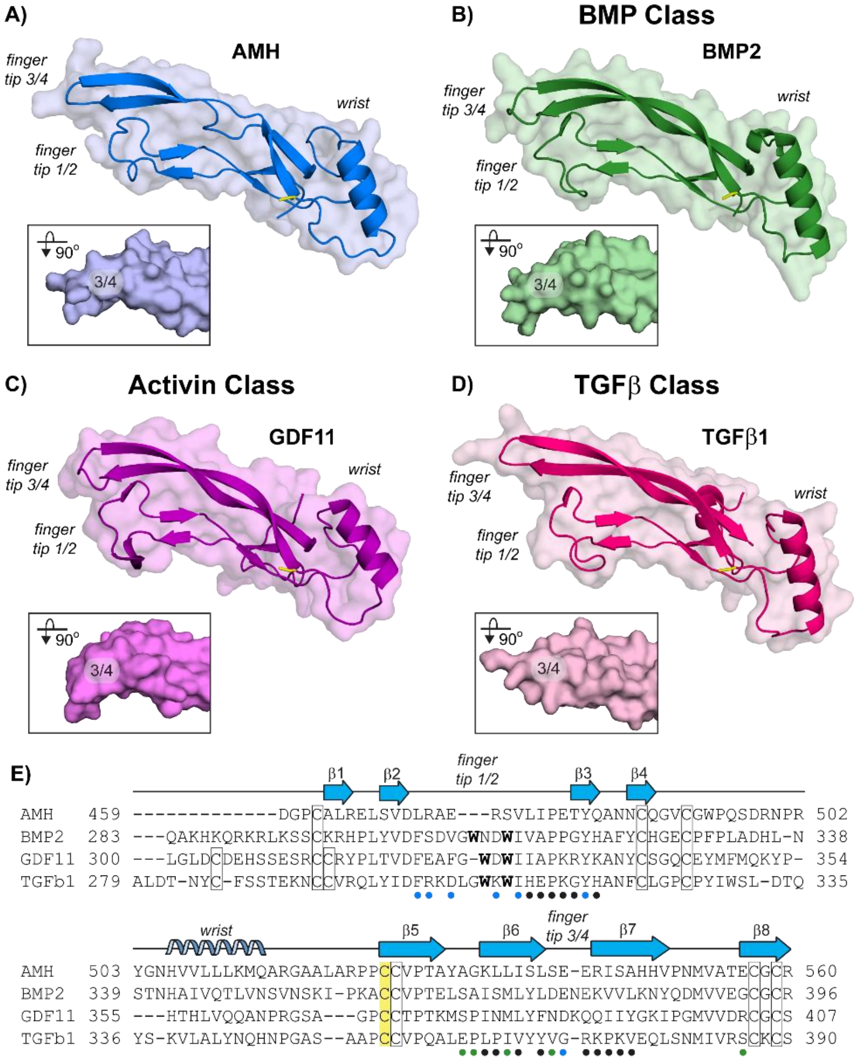

Figure S5. Ligand comparison. Structure of ligand monomers for AMH **(A)**, BMP (BMP2-PDB ID 2GOO) **(B)**, Activin (GDF11-PDB ID 6MAC) **(C)**, and TGFβ (TGFβ1-PDB ID 3KFD) **(D)** classes are shown in cartoon with fingertip 1/2, fingertip 3/4, and wrist labeled. Box shows close up of fingertip 3/4 rotated 90 from monomer figure. **E)** shows sequence alignment of ligands with secondary structural features labeled based on AMH structure. Cysteines are labeled by black boxes with the cysteine involved in dimerization highlighted in yellow. Conserved Trp (W) residues in type I binding site are bolded. Dots below sequence show residues used by either AMH (blue), BMP2 (green), or both AMH and BMP2 (black) to bind to their respective type II receptors, AMHR2 or ActRIIA.
